# Do Breast Cancer Patients Manage to Participate in an Outdoor, Tailored, Physical Activity Program during Adjuvant Breast Cancer Treatment, Independent of Health and Socio-Demographic Characteristics?

**DOI:** 10.3390/jcm11030843

**Published:** 2022-02-05

**Authors:** Ragna Stalsberg, Gro Falkenér Bertheussen, Harriet Børset, Simon Nørskov Thomsen, Anders Husøy, Vidar Gordon Flote, Inger Thune, Steinar Lundgren

**Affiliations:** 1 Department of Circulation and Medical Imaging, Norwegian University of Science and Technology, N-7491 Trondheim, Norway; 2 Department of Neuromedicine and Movement Science, Norwegian University of Science and Technology, N-7491 Trondheim, Norway; gro.f.bertheussen@ntnu.no; 3 Department of Physical Medicine and Rehabilitation, St. Olav Hospital, Trondheim University Hospital, N-7030 Trondheim, Norway; 4 Clinic of Surgery, St. Olav Hospital, Trondheim University Hospital, N-7030 Trondheim, Norway; harriet.borset@stolav.no (H.B.); steinar.lundgren@ntnu.no (S.L.); 5 Centre for Physical Activity Research, Rigshospitalet—Copenhagen University Hospital, DK-2100 Copenhagen, Denmark; simon.noerskov.thomsen@regionh.dk; 6 Department of Sports Medicine, Norwegian School of Sport Sciences, N-0806 Oslo, Norway; andershu@nih.no; 7 Department of Oncology, Oslo University Hospital, N-0424 Oslo, Norway; uxflov@ous-hf.no (V.G.F.); ithune@ous-hf.no (I.T.); 8 Institute of Clinical Medicine, University of Oslo, N-0316 Oslo, Norway; 9 Department of Clinical and Molecular Medicine, Norwegian University of Science and Technology, N-7491 Trondheim, Norway

**Keywords:** breast cancer, physical activity, adherence, withdrawal, sociodemographic, outdoors intervention

## Abstract

Exercise could reduce the side-effects of adjuvant breast cancer treatment; however, socio-demographic, health, and intervention conditions may affect patients’ adherence to interventions. This study aimed to examine adherence to a 12-month outdoor post-surgery exercise program among newly diagnosed breast cancer patients during adjuvant treatment, and to identify socio-demographic and health-related predictors. In total, 47 women with invasive breast cancer stage I–II or ductal/lobular carcinoma grade 3 were included pre-surgery and randomized two weeks post-surgery to exercise (2 × 60 min/week). Patient characteristics (body-mass index (BMI), socioeconomic status, comorbidity, physical activity, and maximal oxygen uptake (VO2max)) were recorded pre-surgery. Correlations between adherence and patient characteristics and statistics for between-group differences were performed. The mean age was 54.2 years, mean BMI 27.8 kg/m^2^, and 54.2% received chemotherapy. Completers had a mean adherence of 81%, independent of season. Withdrawals (23%) occurred after a mean of 6.5 weeks (0–24 weeks), they were suggestively older, had lower socioeconomic status and pre-surgery VO2max, and higher BMI. Household income was significantly lower among withdrawals. There were insignificant correlations between adherence and health conditions. High adherence is achievable in a Nordic outdoor physical exercise program in breast cancer patients during adjuvant treatment, including chemotherapy. Additional studies are needed to clarify follow-up needs in some groups.

## 1. Introduction

Physical activity (PA) has consistently been observed to reduce the risk of postmenopausal breast cancer [1,2,3]. In addition, previous studies indicate that PA during adjuvant breast cancer treatment may reduce unfavorable side-effects [4] and suggestively reduce recurrence and increase survival [5,6,7,8]. Recent PA intervention trials, and qualitative studies, support that PA may improve physical fitness [1,6,7,8], physical functioning [7,9,10], fatigue [1,7,10,11,12], and quality of life [1,7,10] in breast cancer patients. However, the effect of PA in a clinical PA intervention depends on how the intervention is designed as part of breast cancer rehabilitation (i.e., time to start, type, intensity and doses of PA), as well as the patients’ opportunity to participate, considering their sociodemographic characteristics and health conditions. Hence, to translate research results into clinical recommendations and practice, knowledge about whether specific PA intervention designs are justifiable and feasible for patients with different health and sociodemographic characteristics is needed.

Adherence may be defined as “the extent to which a person’s behavior […] corresponds with agreed recommendations from a health care provider” [13] and has been a challenge in PA intervention studies [14]. Adjuvant breast cancer treatment may be challenging [6] and give side-effects such as nausea, fatigue, hair loss and chills [7], which can make maximal PA intervention adherence difficult [1]. In addition, the mental strain of being diagnosed with breast cancer [10], medical complications, deterioration of medical condition, personal or social problems may affect how well patients adhere [11]. Moreover, because patients are more susceptible to infections during adjuvant breast cancer treatment, for them to accomplish the types and doses of PA needed to achieve the intended effect, the interventions must be performed within secure and trustworthy settings, also regarding the risk of infection.

Previous trials involving PA in breast cancer patients vary in settings that influence the adherence rates. For example, shorter interventions (3–6 months) have predicted higher adherence than longer interventions (1–2 years) [15], as do home-based exercise compared to center-based programs for patients >50 years [16], supervised compared to unsupervised PA programs, and less physically demanding interventions compared to trials involving strenuous PA [17]. Several studies support that 12-month exercise programs for patients result in higher maintenance of physical benefits compared to 6-month intervention programs [18]. According to these studies, the length of the intervention may thus help make exercise become part of patients’ lifestyle, which in turn potentially may improve quality of life, and influence breast cancer recurrence and survival [19,20]. 

Most studies reporting adherence to supervised exercise interventions in breast cancer patients during adjuvant treatment include indoor training. In addition, previous PA interventions that are conducted in part outdoors, are either of lower intensity (walking programs) or have reported cancelled sessions due to bad weather [21]. If we consider the risk of infection during the treatment period and add the importance of physical distance learned during the COVID-19 pandemic, adopting an outdoor design might be a sound alternative design. Adding the perceived positive health effect of being in nature [22] and the possible relief of departing from hospital settings, it is valuable gaining knowledge about whether an outdoor PA intervention is feasible for breast cancer patients.

However, there are reasons to believe that there are health or sociodemographic differences among patients affecting adherence to exercise programs. Not least is high socioeconomic status, which is associated with more leisure-time PA and exercise in general [23]; thus, exercise programs may not fit all equally well. Nevertheless, only a few randomized PA trials during breast cancer treatment have assessed adherence across socioeconomic status [24,25,26,27,28]. Still, higher educational level seems to predict higher adherence to PA in some breast cancer populations [17,26], and being employed has been associated with better intensity of PA adherence [25]. In some studies, socioeconomic differences between completers and withdrawals are found to be either non-existent [28,29], or dropouts have been reported to be unemployed or have lower education [27]. Moreover, some health factors, which also may be related to socioeconomic status, are reported to reduce adherence to PA, as follows: higher body mass index (BMI)), sedentary baseline behavior [30], higher levels of fatigue [31] and low lower-body muscle strength at baseline [24]. Further, higher adherence has been associated with breast cancer patients who were fitter [24,30], less depressed [16] and interested in exercise [25]. In order to maintain socially equal health care services, more knowledge on possible socioeconomic and health-related differences in adherence to PA interventions is needed.

In addition to health and sociodemographic variables, the type of treatment may be of importance for breast cancer patients’ adherence to a PA-intervention; receiving radiotherapy in addition to chemotherapy have predicted low attendance to supervised exercise [17], yet in a short-term home-based walking program, receiving chemotherapy was associated with better adherence compared to adherence when receiving radiotherapy [32]. Interestingly, in a previous 12-month exercise trial, more breast cancer patients who had received neoadjuvant chemotherapy had high adherence to the supervised exercise (≥67% attendance) compared to patients who had received adjuvant or no chemotherapy [24].

Although no golden standard of “good adherence” to PA interventions in breast cancer patients has been established, due to inconsistent methods of measurement across studies, thorough knowledge about which patients have difficulties in participating in an outdoor clinical PA intervention is essential when it comes to who needs extra follow-up during treatment and rehabilitation. Thus, the main aim of the present study was to investigate adherence to an outdoor 12-month post-surgery supervised exercise intervention during seasonal variation among newly diagnosed breast cancer patients receiving adjuvant treatment, and to identify sociodemographic and health-related adherence predictors.

## 2. Materials and Methods

### 2.1. Participants and Study Design

In a larger multicenter study, women aged 18–75 years, with ductal/lobular carcinoma in situ (DCIS grade 3/LCIS) or invasive BC stage I–II were invited to participate in a prospective two-armed 12-month physical exercise intervention, randomized 10 +/− 2 days after surgery (ClinicalTrials.gov accessed on 16 September 2014, Registration No. NCT02240836). Although attendance was registered similarly at all training locations, complete data were made available from one study center only. Therefore, only patients enrolled at the St. Olav Hospital, Trondheim, Norway, between September 2014 and June 2017 were involved in the present study. Inclusion required Norwegian language skills and no functional or practical limitations. Exclusion criteria were related to musculoskeletal diseases or injuries that make physical activity difficult, not being able to participate (severe illnesses, BMI < 18.5 kg/m^2^ and >40 kg/m^2^, previous bariatric surgery), or with a travel distance > 1.5 h from home to study site.

**Figure 1:**
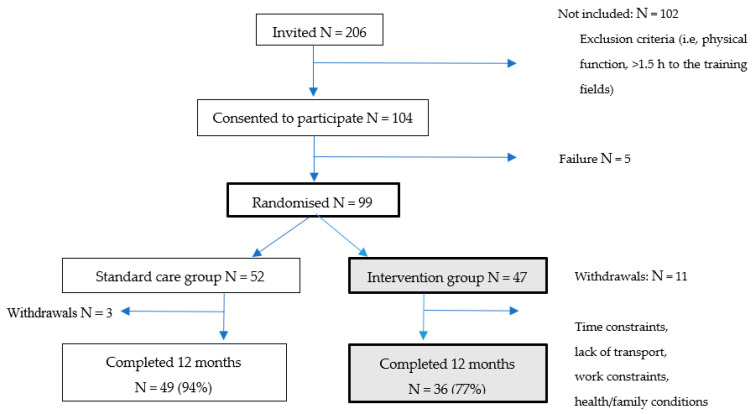
Flowchart of participants through the study.

After baseline assessment and surgery, participants were randomly allocated 1:1 to either the intervention or control group, stratified by menopausal status. In total, 47 patients were enrolled in the intervention (Figure 1).

Data on the efficacy of the physical activity will be reported elsewhere.

### 2.2. The Exercise Intervention

The 12-month outdoor post-surgery group exercise program started two weeks post-surgery and was in accordance with national and international exercise expertise [33,34,35] and included aerobic training of moderate-to-high intensity as well as stretching and weight-bearing activities. Every session was led by specially trained physiotherapists and included 8–12 participants, lasted 60 min and took place two times per week. The exercise program consisted of a 10 min warm-up (intensity corresponding to 60–80% of maximal heart rate (HRmax) or 9–12 on the Borg scale [36]), endurance session (interval uphill of 2 min × 3; HRmax > 80%, Borg scale 15–17), strength session focusing on lower extremities and upper body (8–12 repetitions × 2) and ended with mobility, posture and balance exercises and calming down. Physicians visited once a month to be available for questions regarding exercise, and challenges related to the BC diagnosis and treatment. All group sessions took place outdoors in the forests and fields in an unstable, wet and quite cold climate. Only occasionally, if participants were prevented from group participation, the participants performed similar exercise on their own, and it was then registered as a completed session. A total of eight weeks of absence due to vacations during the 12-month intervention was accepted.

The intervention was free of charge for the patients.

### 2.3. Assessment o f Patient Characteristics and Baseline PA

Baseline variables were assessed pre-surgery by questionnaire (self-reported and interview) (PA, sociodemographic variables) or measurement (BMI, Maximal oxygen consumption (VO2max; mL × kg^−1^ × min^−1^)). Height and weight were measured when wearing light clothing and no footwear; height to the nearest 0.5 cm, and weight to the nearest 0.1 kg on an electronic scale and converted into BMI (kg/m^2^). VO2max were assessed by means of a modified Balke treadmill protocol on a Woodway treadmill (Weil am Rhein, Germany). VO2max was calculated as the average of three highest sequential 10 s intervals, measured at pre-surgery.

Socioeconomic status was measured as total years of education and highest level of education (elementary school; vocational training; high school; college/university degree ≤ 4 years; university degree > 4 years; other), occupational class (see [37]), and household income (NOK 1 ≈ EUR 0.1) (<350,000; 350,000–599,999; 600,000–999,999; ≥1,000,000)

Baseline PA level was based on the last 12 months of occupational PA (heavy manual work, frequently lifting and walking, frequently walking/sedentary) and leisure time PA (previously validated, see [38]). The total sum of minutes of all reported leisure-time PA (min/year = min/bout × bouts/week × (months/year × 4.3 weeks/month) will, for a woman who swam 2–3 times/week, 40 min/bout, 4 months/year, hiked > 4 times/week, 60 min/bout, 12 months/year, get a score of 2.5 × 40 × (4 × 4.3) + 4.5 × 60 × (12 × 4.3) = 15,652 min/year (261 h/year). All data from questionnaires were manually checked for inconsistencies (i.e., missing or obviously erroneous data plots) by 2 researchers and the study nurse during plotting and quality checks. Comorbidity was self-reported, but also checked and registered by the same trained study nurse at the baseline interview and reported as total number of comorbidities.

### 2.4. Breast Tumour Characteristics and Patient Treatment

The excised tumors were characterized histologically and immunohistochemically and classified according to TNM, histological type, grade and receptor status, as described [39]. Axillary lymph nodes status was reported as number of affected and removed lymph nodes and reported as positive (pN+) or negative (pN0) status. Dependent on patient and tumor characteristics, chemotherapy was given in accordance with current national treatment guidelines in accordance with international guidelines (www.nbcg.no accessed on 31 December 2021); The adjuvant chemotherapy started 4–6 weeks post-surgery and lasted for 12–24 weeks. Post-surgery daily radiation therapy for 3–5 weeks was started 3–4 week after end of chemotherapy, otherwise 5 weeks post-surgery. Nine participants received intraoperative radiotherapy (IORT) at time of primary surgery. Endocrine therapy was started after end of chemotherapy, or 3–4 weeks post-surgery for those who did not need chemotherapy.

### 2.5. Analyses 

Adherence to overall group exercise was referred to as a percentage of full attendance, defined as attending 80 group sessions during 12 months, excluding holidays and is in accordance with regular working/school days. Quarterly adherence rates were based on participants’ number of attendances each quarter divided by the maximum number of exercise sessions possible during a quarter. 

Possible seasonal adherence variations were first visualized by means of colored plots for attendance and non-attendance in Excel, following the week of the year (starting with January), and collated with the four seasons (winter: December–February, spring: March–May, summer: June–August, fall: September–November), which vary in average temperature (+2 °C (winter)–+19 °C (summer)) and in average rainfall (4.65 cm (spring)–8.99 cm (fall)). Monthly attendances were then summarized to identify seasonal variations.

Participants who either did not meet or withdrew from the group sessions during the first six months are reported as withdrawals (*n* = 11). The remaining participants were treated as completers (*n* = 36).

The relationship between sociodemographic variables and adherence was examined using the Kendall’s tau-b and the Pearson’s r correlation coefficient method for ordinal or scale and nominal measures, respectively. A Mann–Whitney U test and Kruskal–Wallis test were employed to examine distribution differences in adherence between groups. For the Mann–Whitney U test and the Kruskal–Wallis test, the variables were recoded into high/low education level (≥college degree/<college degree); white-collar/blue-collar occupation (see [37] for details); income level (NOK 1 ≈ EUR 0.1) (low: ≤599,999/medium:–999,999/high: ≥1,000,000); lower/higher age (29–52/53–75); active/sedentary occupational PA (heavy manual, lifting and walking, walking/sedentary); and active/sedentary leisure-time PA (>150 min/week/<150 min/week).

The level of significance was set at 0.05 (*p* ≤ 0.05). Holm–Bonferroni corrections for multiple tests were performed on significant correlations, and Bonferroni corrections as post hoc Kruskal–Wallis tests were performed to detect significant differences in mean rank adherence between groups. Omitted data for variables other than adherence were treated as missing and accordingly decreased sample size. SPSS Statistics (v25) was applied for statistical analyses. 

## 3. Results

The participants were on average 54.2 years and 55% had ≥college degree, 55% had white-collar occupations, and 28% belonged to the highest income group. Mean BMI was 27.8 kg/m^2^ and mean VO2max was 28.4 mL × kg^−1^ × min^−1^. On average, the participants reported leisure-time PA close to 3 h/week (all intensities included) the preceding year pre-diagnosis. A total of 68% of the participants reported co-occurrences of two to five other diseases, whereas 38% reported one disease in addition to BC. A total of 70% had breast-conserving surgery (BCS). Endocrine therapy, chemotherapy, trastuzumab and radiotherapy were given to 64, 53, 19 and 77%, respectively (Table 1).

### 3.1. Withdrawals versus Completers

**Figure 2:**
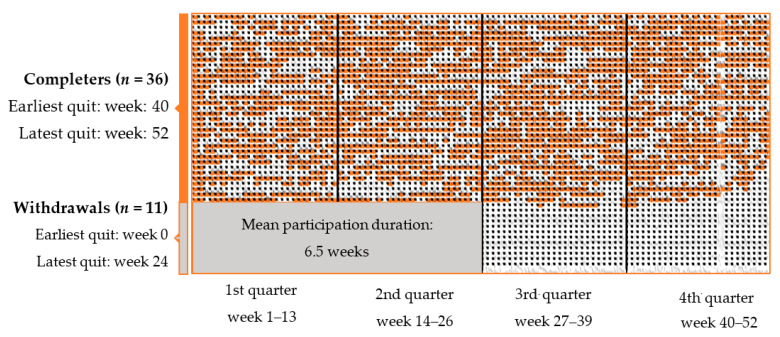
Attendance from the start throughout the last group exercise session attended. Orange plots indicate attendances. Each row represents one patient. Grey rectangle indicates the group average participation period of the withdrawals (in order to avoid patient recognition).

Out of the 206 patients invited, 102 were not able or interested in participating due to transport difficulties, travel distance >1.5 h, workplace constraints, and for some, combined with BC treatment, participating in group exercise was too burdensome. Of the 47 patients who consented to participate and randomized to the intervention group, 11 patients withdrew within 6 months after start-up due to health/family conditions, transport, workplace constraints or lack of time (Figure 2). No withdrawals were due to resection. 

Among withdrawals (*n* = 11), the mean age was 58 years, and 36% had a college degree or more, 54% were in a white-collar position, and 27% was in the highest income group. On average, pre-trial leisure-time PA/week (all intensities) was reported to be 3.5 h, BMI was 29.7 kg/m^2^, and Vo2max 24 mL × kg^−1^ × min^−1^. Compared with the completers (*n* = 36), the withdrawals as a group appeared to be older, have lower socioeconomic status, higher BMI, and lower VO2max, despite more minutes in pre-trial leisure-time PA. Both education level and household income, as well as occupational class, were on average lower among withdrawals than among completers.

Chi-square tests of independence showed that the difference was statistically significant only for household income (*p* = 0.005) (Table 1). Mann–Whitney U tests indicated that pre-trial leisure-time PA were (non-significantly) higher for withdrawals (mean rank = 19.9) than completers (mean rank = 25) (U = 108, *p* = 0.269). The baseline BMI was (non-significantly) lower among withdrawals (mean rank = 22.8) compared to completers (mean rank = 28) (U = 154, *p* = 0.279). An independent sample t-test showed a significantly lower pre-surgery VO2max in withdrawals (M = 24.0, SD = 7.89) compared to completers (M = 29.7, SD = 5.4); t (45) = −2.69, *p* = 0.010 (Table 1).

### 3.2. Adherence Rates

**Figure 3:**
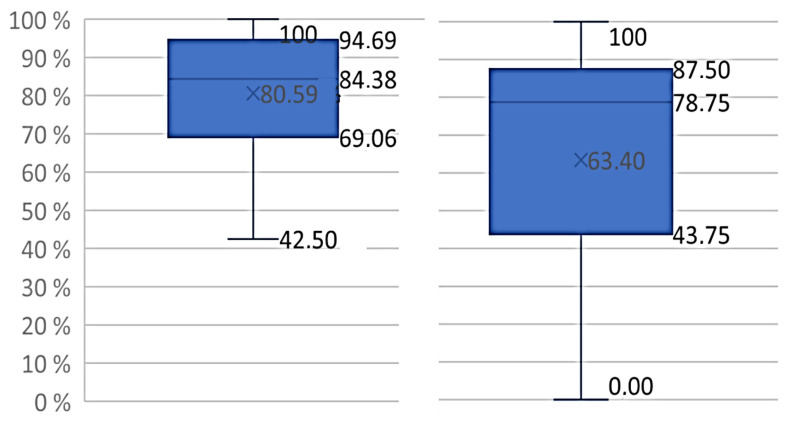
Total attendance to group exercise sessions (%). Left: exercise group; completers (*n* = 36); right: exercise group; total (*n* = 47).

Mean adherence rate was 81% (median = 85.4) among completers (*n* = 36) and 63% (median = 78.8%) for the total intervention group (*n* = 47) (Figure 3). For completers, the shortest individual participation period duration from start to finish, independent of adherence rate, was 40 weeks. In the group of withdrawals (*n* = 11), the longest individual participation period duration was 24 weeks (Figure 2). Variations in total attendances to exercise sessions throughout the seasons of a year, including data from all participants added up (i.e., the sum of all ongoing intervention periods from 2014 to 2017) showed that attendance rates dropped during the following weeks of holidays: July and August, and December and January had pronounced drops in attendance, April had a minor decline. The highest number of attendances was observed in November, whereas the fewest was in August. For completers, only small differences (60–63%) in mean adherence after each quarter were observed, regardless of which time of the year the participant was included.

### 3.3. Associations between Adherence and Baseline Variables 

No data of socioeconomic status correlated significantly with adherence to group exercise at any quarter (Table 2). Among completers (*n* = 36), age correlated positively with adherence after 9 (r = 0.341, *p* = 0.042) and 12 months (r = 0.0366, *p* = 0.028), and with adherence relative to 80 group sessions (r = 0.369, *p* = 0.027). Number of comorbidities showed a weaker negative, but significant correlation with adherence in the first quarter (r = −0.237, *p* = 0.04) when withdrawals were included (*n* = 47) in the analyses. However, none of these associations remained significant after Holm–Bonferroni corrections. In the sample of completers, neither VO2max, BMI or number of comorbidities correlated significantly with adherence at any quarter of the intervention period.

Although weak and non-significant trends, among completers, there was a positive correlation between adherence and level of pre-trial leisure-time PA in the preceding 12 months. The correlation between adherence and type of pre-trial occupational PA decreased from the first through the third intervention quarter. When withdrawals were included in the analyses, a similar trend was seen for type of occupational PA (higher adherence, more active occupation); however, the correlation was weaker and turned negative for leisure-time PA.

The non-parametric tests of distribution differences in adherence for different baseline variables did not produce any results which could be interpreted as credible evidence of real differences in adherence between groups (Appendix A).

## 4. Discussion

To our knowledge, this is the first study to report adherence to a 12-month outdoor exercise program in newly diagnosed breast cancer patients starting early post-surgery and ongoing during adjuvant treatment in a climate with pronounced seasonal variations. The overall mean adherence to the present exercise intervention was 81% when withdrawals were excluded from the analysis, and 63% for the total intervention group, withdrawals included. Previous exercise interventions in breast cancer patients report adherence rates ranging from 42–91% [40], 71–83% [41], and 70–95% [21,26,31,42,43]. Unfortunately, the comparability between studies is limited, due to inconsistent operationalizations and calculations of adherence [40,44], variations in intervention designs, such as the ambient conditions, including climate and weather, as well as the duration and timing of the exercise program in the post-surgery period. Unstandardized adherence measures, different intervention designs, and the fact that patients differ in terms of physical fitness at baseline, prevent us from establishing which adherence rate would be sufficient for a PA intervention to be effective. Indeed, studies support that 12-month exercise programs for breast cancer patients results in physical benefits compared to a 6-month exercise program [18]. The length of the intervention might thus help make exercise part of patients’ lifestyle, which could potentially improve quality of life, influence recurrence of breast cancer and survival in the long run [20]. However, how high adherence needs to be in order to succeed, is complex and dependent on a multiple of variables.

### 4.1. Adherence and Intervention Design

Quarterly adherence rates came out virtually equal, and the trend of attendances was stable throughout the 12 months. These results are in contrast to the suggestion that shorter PA interventions (3–6 months) are more feasible in terms of adherence rates compared with longer trials (1–2 years) [15]. Basically, besides demonstrating stability, our results indicate that the time span of the intervention may be of less importance in explaining adherence. However, considering that the participation among withdrawals ended in the 24th week, a shorter intervention period may have resulted in higher retention rates.

A feature of our study was the regular and professional support provided by experienced physiotherapists and physicians. As suggested in previous studies [24,45], counselling increases the motivational readiness to adhere to PA guidelines among breast cancer patients burdened with side-effects and medical appointments. Other studies also highlight continuous attention as a crucial factor in PA interventions [31]. Thus, the pre-intervention physical testing, professional presence and follow-up, in addition to the group identification and internal mutual support from exercise group members, may have positively affected the stability in group exercise attendance in our study (Figure 3).

As the Nordic climate is characterized by rainy days and harsh winters, the outdoor exercise design could have made the adherence rates more vulnerable to seasonal variations compared to the invariant indoor exercise conditions applied in most studies previously mentioned. The climate has been suggested as an influence on adherence even across indoor training venues, due to a geographical split [46], and bad weather has previously caused cancellations of training sessions [21]. However, in our study, seasonal attendance variations seem to coincide with national standard holiday periods rather than changing weather conditions. Such a finding further supports the effect of professional follow-up and group dynamic suggested above, and also the feasibility of an outdoor group exercise intervention, regardless of climate and seasons, and with the benefit of low costs compared with indoor exercise program where rental costs are added.

### 4.2. Socioeconomic Status

The withdrawals had lower average socioeconomic status, including lower household income, compared to completers (Table 1). These tendencies support results from previous studies showing that high adherence to PA may be related to higher socioeconomic status [17,26]. The fact that some participants could not afford the transport expenses, had workplace constraints related to being occupied with group exercise within the working hours, or issues related to exemption cards for public health services, could partly explain these associations. Furthermore, as education seems to be positively associated with the leisure-time domain of PA [23], one could also expect that adherence to PA interventions was related to pre-trial PA level [47,48].

However, we found no evidence for a relationship between adherence rate and socioeconomic status in our data, corresponding to previous reports of adherence to PA among BC patients [49,50]. Usually, such statistically insignificant results are interpreted as a lack of credible evidence of real differences in adherence between socioeconomic status groups. Hence, they are perceived as less interesting, and often larger sample sizes are called for. As the sample-size in our study is rather small, such explanations seems reasonable. However, as pointed out in a revived debate in Nature and the BMJ [51,52], a non-significant result is no proof of no difference [53]. Rather than concluding with it being uninteresting, an uncertain result should be considered from alternative angles.

As also suggested by others [49], an alternative interpretation of insignificant socioeconomic status results could be that they relate to sample misrepresentation of socioeconomic status group distributions. In our study, 55% of all participants held >13 years of education, whereas only 17% had <high school education. These numbers diverge from comparable Norwegian statistics of educational levels in equivalent age and patient groups (see [54] for patient group comparisons of educational level, [55] for normal group comparison of educational level, and [56] for normal group comparison of income level). Although the association between higher educational level and risk of breast cancer is evident [54,57,58], these numbers show that neither is the present study sample representative in terms of socioeconomic status group distribution. Thus, a statistical test of socioeconomic differences would in fact be less valid to the population. Previous evidence shows that individuals of lower socioeconomic status less often participate in research projects compared to individuals with higher socioeconomic status [59,60]. This, in addition to the fact that breast cancer patients in low socioeconomic groups fail to meet inclusion criteria in PA studies, due to more advanced cancer at diagnosis, and that exercise interventions unfortunately do not fit all social groups [61,62,63], makes it likely that previous studies of adherence to PA interventions also suffer from sample biases similar to the one identified in the present study. In other words, the lack of statistically significant differences between socioeconomic groups identified in previous analyses may stem from small study samples, and, not least, that any subdivisions of high and low socioeconomic status may be erroneous in terms of group characteristics. Unfortunately, because many articles lack information on sociodemographic distributions within the study samples, representativeness (hence the real association between socioeconomic status and adherence), is often difficult to decide [64].

Finally, although it is highly probable that differences in data distribution between groups in our study appeared by chance (Appendix A, Table A1), and despite the challenges of representativeness discussed above, one could speculate whether the tendency that high socioeconomic status groups had higher mean rank distribution of attendance to group exercise in the early quarterly compared to later phases might relate to the likelihood of returning to work after breast cancer. Return to work is previously reported to be associated with high education and higher income level [65]. On the other hand, in addition to being statistically non-significant, such changes in bivariate associations over time may reflect the phenomenon of ‘regression toward the mean’ [66], rather than real changes in adherence over time.

### 4.3. Health-Related Variables

Our analyses of associations between health variables and adherence rendered uncertain findings; however, age may have influenced on study participation in two ways. First, the group of withdrawals had higher average BMI and reported more pre-trial minutes of leisure-time PA compared to completers, but significantly lower levels of VO2max (Table 1). The level of VO2max is lower in our population than what is considered reference values for a general population of Norwegian women of same age [67]. However, the fact that withdrawals also had higher mean age than the whole intervention group may explain their lower level of VO2max. In addition, they also prefer low intensity PA before vigorous PA. Previous studies showing that VO2max has a ten-year year decline of about 10% in women [68] and that there may be a progressively increase in BMI with age in women [69], support the interpretation of an interrelationship between age, BMI and VO2max in our data, despite the statistical insignificance.

Secondly, the negative correlation initially found between comorbidity and adherence, could, if statistically significant, be interpreted as the more diseases, the more difficult it is to be physically active. Compared to a Bonferroni correction, the Holm–Bonferroni reduces the power to detect real effects [70]. Therefore, when the negative relationship between comorbidity and adherence were found not significant after Holm–Bonferroni correction (Table 2), we may have failed to acknowledge real associations, and the above interpretations were after all reasonable. This also applies to the positive age—adherence correlation at 9 and 12 months for the group of completers, which was found significant before, but not after Holm–Bonferroni correction (Table 2). Therefore, it might be that some age-related factors influence long-term adherence to interventions, such as ours. Being younger and probably more often reported fit, and also part of the workforce, may imply family or work-related duties that influence on attendance stability (remember that our intervention was arranged within the working hours). On the other hand, being older may hinder some patients from participating in general, probably due to comorbidity. 

### 4.4. Strengths and Limitations

The intervention on which our adherence data are based shows several strengths. Firstly, it was provided as an easily accessible service at low costs, and was conducted in natural outdoors settings, without equipment requirements other than training shoes, comfortable clothing, and walking poles. Along with the fact that the intervention was completed along current clinical practice, this speaks to a possible rapid and uncomplicated implementation of an additional treatment pathway with PA included. Second, it was conducted in secure and trustworthy exercise settings, as the groups were led by trained physiotherapists and regularly visited by experienced physicians. The outdoor setting is safer regarding the risk of getting infectious diseases, which is important as patients undergoing breast cancer treatment may have a weakened immune system. In addition, the small exercise group size made individual adaptations possible, additionally entailing advantageous groups dynamics. Chemotherapy was set up to the day after a group exercise and may have contributed to reduce absence from group sessions. It is reasonable to believe that these factors had positive impact on the ability to accomplish the types and doses of PA necessary to achieve the intended effect. Finally. The COVID-19 pandemic has actualized the importance of focusing on the risk of infection in vulnerable patient groups, making outdoor interventions even more relevant.

However, the small sample size reduced the possibility of determining statistical significance between groups. Second, from the low-cost indicated above, it follows that the participants had to bear the time to travel and costs of transport hampering remotely living patients from participating. Third, we cannot rule out the possibility that a selection bias occurred because the participants were asked to join an exercise study, which may have excluded patients with low pre-trial leisure-time PA. On the other hand, all eligible patients who were diagnosed during the study period were invited to participate, hence reducing the risk of selection bias. Fourth, exercise intensity was not reported. However, the intervention protocol required a certain exercise intensity, thus the lack of specific intensity data was not decisive.

## 5. Conclusions

The adherence rate to our 12-month outdoor supervised group exercise intervention among BC patients in the first year after diagnosis and during cancer treatment was 81%, uninfluenced by the harsh Nordic climate. Regular and professional support provided by experienced physiotherapists and physicians may have increased the adherence to the outdoor intervention design, although adherence probably also relates to higher age. As our study include outdoor exercise at low cost, the results support, but also extend previous findings.

A small sample size, and a sample homogeneity at the expense of lower socioeconomic status groups, challenges subgroup analyses of adherence rates. Such issues act as impediments in identifying groups of patients to whom we need to accommodate our PA interventions. The fact that exercise sessions were conducted during working hours, may have restrained intervention adherence. Further and larger studies are needed to confirm the barriers of comorbidity, fitness, high costs and being occupied by paid work suggested in our study, and also to explore others.

## Figures and Tables

**Table 1:** Baseline characteristics of patients randomized to exercise intervention (*N* = 47, *n* = 36 vs. *n* = 11).

Patient Characteristics at Baseline	*All Patients, n* = 47 M (SD) or N (%)	*Completers, n* = 36 M (SD) or N (%)	*n* = 36 vs. *n* = 11 *p* Value
Age	54.2 (10.1)	53.0 (9.8)	≤55 years; >55 years: 0.427 ^c^
Education			n.a.
*College/university degree > 4 years*	12 (25.5)	8 (22.2)	
*College/university degree ≤ 4 years*	14 (29.8)	14 (33.3)	
*High school = 3 years*	13 (27.7)	9 (19.4)	
*Vocational training/elementary school*	8 (17.1)	5 (13.8)	
Occupation			n.a.
*Management position public/private*	7 (14.9)	5 (13.9)	
*Management position, academic*	6 (12.8)	4 (11.1)	
*Lower profession*	13 (27.7)	11 (30.6)	
*Non-professional occupation*	9 (19.1)	9 (25.0)	
*Self-employed business/skilled, artisan*	6 (12.8)	4 (11.1)	
*Semi-skilled, unskilled*	5 (10.6)	2 (5.6)	
Household income ^a^			0.005 *^c^
*High*	13 (27.7)	10 (27.8)	
*Medium*	17 (36.2)	16 (44.4)	
*Low*	17 (36.2)	10 (27.8)	
Currently smoking	7 (14.9)		n.a.
Number of comorbidities	1.2 (1.2)	5 (13.9)	n.a.
Occupational physical activity ^b^			0.909 ^c^
*Sedentary*	22 (46.8)	18 (50.0)	
*Frequently walking*	8 (17.0)	7 (19.4)	
*Frequently walking and lifting/heavy*	10 (21.2)	8 (22.2)	
Leisure time physical activity (min/year) ^b^	8477 (6419)	7710 (5615)	0.269 ^d^
BMI, kg/m^2^	27.8 (5.5)	27.3 (5.2)	0.279^d^
VO2max mL × kg^−1^ × min^−1^	28.4 (6.4)	29.7 (5.4)	0.010 *^,e^
Tumour characteristics	N (%)	N (%)	*p* value
Histology			0.674 ^c^
*IC*	44 (93.7)	34 (94.6)	
*DCIS /LCIS only*	3 (6.4)	2 (5.6)	
Pathologic tumour size, mm			0.810 ^c^
*≤10*	10 (21.3)	7 (19.4)	
*10–20*	25 (53.2)	20 (55.6)	
*>20*	12 (25.5)	9 (25.0)	
Grade			0.630 ^c^
*Grade 1*	11 (23.4)	8 (22.2)	
*Grade 2*	19 (40.4)	16 (44.4)	
*Grade 3*	14 (29.8)	10 (27.8)	
*ND, DCIS/ LCIS*	3 (6.4)	2 (5.6)	
Lymph node involved			0.336 ^c^
*0*	33 (70.2)	24 (66.7)	
*1–3*	13 (27.7)	11 (30.6)	
*>3*	1 (2.1)	1 (2.8)	
ER/PgR status			0.059 ^c^
*Positive*	41 (87.2)	33 (91.7)	
*Negative*	3 (6.4)	1 (2.8)	
*ND, DCIS/LCIS*	3 (6.4)	2 (5.6)	
HER 2 status			0.172 ^c^
*Positive*	11 (23.4)	10 (27.8)	
*Negative*	34 (72.3)	24 (66.7)	
*ND, DCIS/ LCIS*	2 (4.3)	2 (5.6)	
Treatment	N (%)	N (%)	*p* value
Surgery (mastectomy/BCS)	14 (29.8)/33 (70.2)	12 (33.3)/24 (66.7)	0.336 ^c^
Endocrine therapy	30 (63.8)	26 (72.2)	0.030 *^,c^
Chemotherapy *	25 (53.2)	21 (58.3)	0.201 ^c^
Trastuzumab	9 (19.1)	9 (25.0)	0.065 ^c^
Radiotherapy	36 (76.6)	27 (75.0)	0.640 ^c^
Zolendronic acid	17 (36.2)	14 (38.9)	n.a.

^a^ High: NOK ≥ 1,000,000; Medium: NOK 600,000-999,999; Low: NOK <350,000–599,999 ^b^ Numbers may vary due to missing information. ^c^ Chi-square test, ^d^ Mann–Whitney U-test, ^e^ T-test. * Statistically significant, α = 0.05. Abbreviations: IC, invasive carcinoma; DCIS, ductal carcinoma in situ; LCIS, lobular carcinoma in situ; ER, estrogen receptor; PgR, progesterone receptor; HER2, human epithelial receptor; IORT, intraoperative radiotherapy; ND, not done; BMI, body mass index (kg/m^2^); n.a., not assessed. * fluorouracil, epirubicine and cyclophosphamide (FEC) or epirubicine and cyclophosphamide (EC), every third week for 4–6 cycles, or 4 cycles of either FEC or EC, and/or Taxanes (every third or weekly) for 12 weeks.

**Table 2:** Correlations between sociodemographic and clinical characteristics of the patients, by adherence to supervised group exercise program (60 min × 2/week)—overall, and stratified by four periods (1st, the 2nd, the 3rd, and the 4th quarter).

Variable	1st Quarter	2nd Quarter	3rd Quarter	4th Quarter	80 Group Sessions
Completers in exercise group (n = 36)					
^a^ *Total years of education*	0.093	−0.036	−0.088	−0.205	−0.195
^a^ *Occupation class*	−0.026	−0.062	−0.031	0.042	0.067
^a^ *Household income*	−0.206	−0.179	−0.103	−0.126	−0.106
^a^ *Occupational activity*	0.226	0.150	0.039	0.090	0.091
^a^ *Leisure-time physical activity*	0.247	0.158	0.070	0.045	0.059
^b^ *Age*	0.210	0.236	0.341 *	0.366 *	0.369^*^
^b^ *BMI (body weight/body height^2^)*	−0.229	−0.236	−0.155	−0.117	−0.085
^b^ *Baseline VO2max*	−0.259	−0.110	−0.154	−0.052	−0.067
^a^ *Comorbidities*	−0.168	−0.116	−0.050	0.015	−0.004
Withdrawals included (n = 47)					
^a^ *Total years of education*	0.024	−0.042	−0.054	−0.120	−0.114
^a^ *Occupation class*	0.051	0.002	−0.020	−0.014	0.027
^a^ *Household income*	−0.082	−0.036	0.054	0.049	0.061
^a^ *Occupational activity*	0.245	0.173	0.054	0.081	0.080
^a^ *Leisure time physical activity*	0.008	−0.078	−0.156	−0.171	−0.159
^b^ *Age*	−0.143	−0.150	−0.119	−0.109	−0.103
^b^ *BMI (body weight/body height^2^)*	−0.098	−0.129	−0.139	−0.148	−0.139
^b^ *Baseline VO2max*	0.038	0.163	0.226	0.287	0.283
^a^ *Comorbidities*	−0.237 *	−0.052	−0.207	−0.172	−0.132

^a^ Tau-b; ^b^ Pearson’s r.* Statistically significant before a Holm-Bonferroni correction, but not after (*p* > 0.05).

## Data Availability

The datasets used and/or analyzed during the current study are available from the corresponding author on reasonable request.

## Appendix A

**Table A1:** Mean rank adherence to group exercise after the 1st, after the 2nd, after the 3rd, and after the 4th intervention quarter (*n* = 36 and *n* = 47). H- and U-statistics for Mann–Whitney U- and Kruskal–Wallis’ tests, respectively (*p*-value).

		1st Quarter		2nd Quarter		3rd Quarter		4th Quarter	
Variable	*n*	Mean Rank	U/H *p* Value	Mean Rank	U/H *p* Value	Mean Rank	U/H *p* Value	Mean Rank	U/H *p* value
Education (*n* = 36)									
<College degree	14	17.32	170.5 (0.59) ^a^	17.93	162 (0.81) ^a^	19.25	143.5 (0.73)^a^	20.50	126 (0.37) ^a^
≥College degree	22	19.25		18.86		18.02		17.23	
Education (*n* = 47)									
<College degree	21	22.19	311 (0.41) ^a^	22.21	310.5 (0.42) ^a^	22.57	303 (0.52) ^a^	23.33	287 (0.76) ^a^
≥College degree	26	25.46		25.44		25.15		24.54	
Occupation (*n* = 36)									
Blue-collar	15	16.17	122.5 (0.36) ^a^	16.0	120 (0.33) ^a^	16.53	128 (0.47) ^a^	17.47	142 (0.80) ^a^
White-collar	20	19.38		19.5		19.10		18.40	
Occupation (*n* = 47)									
Blue-collar	19	21.97	227.5 (0.65) ^a^	21.79	224.0 (0.59) ^a^	22.16	231 (0.71) ^a^	23.08	245 (0.96) ^a^
White-collar	26	23.75		23.88		23.62		22.89	
Income (*n* = 36)									
Low	10	21.65	2.342 (0.31) ^b^	22.00	1.695 (0.42) ^b^	21.65	1.424 (0.49) ^b^	22.30	1.956 (0.37) ^b^
Medium	16	18.97		17.81		16.59		16.41	
High	10	14.60		16.10		18.40		18.05	
Income (*n* = 47)									
Low	17	24.15	2.165 (0.33) ^b^	23.71	1.211 (0.54)^b^	22.56	0.560 (0.75) ^b^	22.74	0.526 (0.76) ^b^
Medium	17	27.12		26.56		25.94		25.91	
High	13	19.73		21.04		23.35		23.15	
Age (*n* = 36)									
≤55years	19	16.50	199.5 (0.23) ^a^	16.97	190.5 (0.36) ^a^	15.87	211.5 (0.11) ^a^	15.50	218.5 (0.07) ^a^
>55 years	17	20.74		20.21		21.44		21.85	
Age (*n* = 47)									
≤55years	23	24.61	262 (0.76) ^a^	24.89	255.5 (0.66) ^a^	23.61	285 (0.85) ^a^	23.26	293 (0.72) ^a^
>55 years	24	23.42		23.15		24.38		24.71	
OPA-level ^c^ (*n* = 36)									
Active	15	19.67	175.0 (0.15) ^a^	18.33	155.0 (0.48)^a^	16.93	134 (0.98) ^a^	17.93	149 (0.63)^a^
Sedentary	18	14.78		15.89		17.06		16.22	
OPA-level ^c^ (*n* = 47)									
Active	18	23.97	206.5 (0.08) ^a^	22.61	236 (0.31) ^a^	20.92	205.5 (0.84) ^a^	21.64	218.5 (0.58) ^a^
Sedentary	22	17.66		18.71		20.16		19.57	
LTPA-level ^d^ (*n* = 36)									
>150 min/week	13	20.08	170 (0.77) ^a^	19.15	158 (0.19) ^a^	17.88	141.5 (0.49) ^a^	17.08	131 (0.77) ^a^
<150 min/week	19	14.05		14.68		15.55		16.11	
LTPA-level ^d^ (*n* = 47)									
>150 min/week	18	22.22	229 (0.56) ^a^	21.42	214.5 (0.84) ^a^	20.86	204.5 (0.94) ^a^	20.31	194.5 (0.74) ^a^
<150 min/week	23	20.04		20.67		21.11		21.54	

^a^ Mann–Whitney U test^;^^b^ Kruskal–Wallis’ test; ^c^ Level of daily occupational physical activity (OPA); ^d^ Minutes of leisure-time physical activity (LTPA)/week, on average, during the last 12 months.
